# Editorial: Sharks and Skates—Ecology, Distribution and Conservation

**DOI:** 10.3390/ani13132222

**Published:** 2023-07-06

**Authors:** Martina Francesca Marongiu

**Affiliations:** Dipartimento di Scienze della Vita e dell’Ambiente, Università degli Studi di Cagliari, Via Tommaso Fiorelli 1, 09126 Cagliari, Italy; mfmarongiu@unica.it

The class Chondrichthyes (sharks, rays and chimeras) is one of the three lineages of fishes and the most evolutionary distinct radiation of vertebrates [1]. It has survived at least five mass extinctions in its 420-million-year history [2,3] and has radiated throughout the major marine (and some freshwater) habitats, dominating upper trophic levels and imposing predation risk in many food webs [4,5].

Chondrichthyes are among the most threatened vertebrates on the planet due to several reasons, such as overfishing, habitat degradation and slow life histories [6,7,8]. According to the International Union for Conservation of Nature (IUCN) Red List criteria, one-third of the world’s chondrichthyan fishes (sharks, rays and chimaeras) are now threatened with extinction [9].

The current observed number of threatened species is more than twice (391 of 1199) [9] that of the first global assessment in 2014, which reported that 181 of 1041 species were threatened [10]. If we assume that Data Deficient (DD) species are threatened in proportion to other species, then over one-third (37.5%) of chondrichthyans are threatened, with a lower estimate of 32.6% (assuming DD species are all Least Concern or Near Threatened) and an upper estimate of 45.5% [9]. Among the three chondrichthyan fish groups, the most threatened are: rays (41% of the 611 assessed species), sharks (35.9% of the 536 assessed species) and chimaeras (9.3% of the 52 assessed species) [9].

The depletion of chondrichthyan populations could lead to worrying ecosystem-level consequences [5,11,12] because many of these fishes are apex or mesopredators that range widely and may affect ecosystem processes through predation and associated risk effects, competition, nutrient transport and bioturbation [13,14,15,16].

In these regards, collecting information about these species is essential to formulate conservation strategies to preserve this precious marine resource.

In this Special Issue, we aimed to expand the knowledge about these important predators, collecting data about the following topics: abundance and distribution, genetic information, life histories (e.g., reproduction, age and growth), demographic analysis, stock assessment and behavior.
